# Ethical use of artificial intelligence to prevent sudden cardiac death: an interview study of patient perspectives

**DOI:** 10.1186/s12910-024-01042-y

**Published:** 2024-04-04

**Authors:** Menno T. Maris, Ayca Koçar, Dick L. Willems, Jeannette Pols, Hanno L. Tan, Georg L. Lindinger, Marieke A.R. Bak

**Affiliations:** 1 Department of Ethics, Law and Humanities, Amsterdam UMC, University of Amsterdam, Amsterdam, The Netherlands; 2 Institute for Healthcare Management and Health Sciences, University of Bayreuth, Bayreuth, Germany; 3 Department of Anthropology, University of Amsterdam, Amsterdam, The Netherlands; 4 Department of Clinical and Experimental Cardiology, Amsterdam UMC, University of Amsterdam, Amsterdam, The Netherlands; 5 Netherlands Heart Institute, Utrecht, The Netherlands; 6 Institute of History and Ethics in Medicine, TUM School of Medicine, Technical University of Munich, Munich, Germany

**Keywords:** Artificial intelligence, Ethics, Sudden cardiac death, Patient values, PROFID, Implantable cardioverter defibrillator, Personalized medicine

## Abstract

**Background:**

The emergence of artificial intelligence (AI) in medicine has prompted the development of numerous ethical guidelines, while the involvement of patients in the creation of these documents lags behind. As part of the European PROFID project we explore patient perspectives on the ethical implications of AI in care for patients at increased risk of sudden cardiac death (SCD).

**Aim:**

Explore perspectives of patients on the ethical use of AI, particularly in clinical decision-making regarding the implantation of an implantable cardioverter-defibrillator (ICD).

**Methods:**

Semi-structured, future scenario-based interviews were conducted among patients who had either an ICD and/or a heart condition with increased risk of SCD in Germany (*n* = 9) and the Netherlands (*n* = 15). We used the principles of the European Commission’s Ethics Guidelines for Trustworthy AI to structure the interviews.

**Results:**

Six themes arose from the interviews: the ability of AI to rectify human doctors’ limitations; the objectivity of data; whether AI can serve as second opinion; AI explainability and patient trust; the importance of the ‘human touch’; and the personalization of care. Overall, our results reveal a strong desire among patients for more personalized and patient-centered care in the context of ICD implantation. Participants in our study express significant concerns about the further loss of the ‘human touch’ in healthcare when AI is introduced in clinical settings. They believe that this aspect of care is currently inadequately recognized in clinical practice. Participants attribute to doctors the responsibility of evaluating AI recommendations for clinical relevance and aligning them with patients’ individual contexts and values, in consultation with the patient.

**Conclusion:**

The ‘human touch’ patients exclusively ascribe to human medical practitioners extends beyond sympathy and kindness, and has clinical relevance in medical decision-making. Because this cannot be replaced by AI, we suggest that normative research into the ‘right to a human doctor’ is needed. Furthermore, policies on patient-centered AI integration in clinical practice should encompass the ethics of everyday practice rather than only principle-based ethics. We suggest that an empirical ethics approach grounded in ethnographic research is exceptionally well-suited to pave the way forward.

**Supplementary Information:**

The online version contains supplementary material available at 10.1186/s12910-024-01042-y.

## Introduction

Artificial intelligence (AI) in medicine holds promise to become one of the main drivers for advancing diagnosis and treatment and improving overall efficiency to meet the ever-rising demand for healthcare services [1]. While AI has made significant progress in areas like medical image analysis in radiology, and natural language processing for extracting and structuring data from electronic health records [2], its general use in medicine is still in its early stages [3]. A research project that aims to introduce the use of AI for clinical risk-prediction in cardiology is the European PROFID project.^1^ With the help of AI-based risk prediction, PROFID aims^2^ to more accurately identify patients who will benefit most from receiving an implantable cardioverter defibrillator (ICD) to prevent sudden cardiac death (SCD). Prescribing ICDs in a more targeted manner than prescribed by current European guidelines might reduce the risk of undertreatment, by preventing SCD among patients at high risk, as well as overtreatment, by mitigating the harms associated with unnecessary implantation [4–8] (See Box. 1). In the Dutch health policy context, this is particularly pertinent, as the Dutch Healthcare Authority recently voiced concerns about the state of Dutch ICD implantation practice [9]. Namely, they signaled a lack of personalized and patient-centered care and advocated for involving patients in medical decision-making in a meaningful way. Several authors of this paper are affiliated with the PROFID project as part of the ethics work package, which aims to examine the ethical issues related to the project. This includes ethical considerations regarding the implementation of an AI-driven prediction model for SCD prevention. For example, what information do patients require to make well-informed decisions when AI-driven risk prediction is used to assess their risk of SCD?

**Box 1:** Clinical context of the PROFID project: Sudden Cardiac Death prevention through ICD-implantation

Sudden cardiac death (SCD) is a significant cause of mortality, accounting for 20% of all deaths in high-income societies [4]. For individuals at increased risk of SCD, the implantable cardioverter defibrillator (ICD) has proven to be an effective intervention, also when implanted prophylactically (primary prevention, i.e., before an SCD-causing cardiac arrhythmia has occurred) [5]. Current European guidelines for ICD implantation for primary prevention of SCD in patients who have previously experienced a myocardial infarction are solely based on the presence of a reduced (< 35%) left ventricular ejection fraction (LVEF) [6]. However, using these guidelines, actual appropriate ICD shocks are only delivered in a small proportion of patients, while SCD mostly occurs in patients who do not meet this eligibility criterion for ICD placement [5]. In other words, under current guidelines there is a discrepancy between patients who receive an ICD and patients who would benefit most from it. Moreover, ICD implantation involves inherent risks for the patient. One in ten ICD patients experiences at least one serious (sometimes potentially life-threatening) complication following implantation, most commonly related to the ICD-leads. These complications can include local and systemic infections and cardiac perforation [7].
Moreover, the fear of receiving a shock and the actual occurrence of both appropriate and inappropriate ICD shocks can lead to prolonged psychological distress [8]. Clearly, there is a need for better prediction of SCD risk in these patients, on which improved guidelines may be based.

With the growing complexity of AI systems and their expanding role in decision-making, both in terms of variety and scale, ethical challenges in the development and use of AI have proven significant [10, 11]. Of these, limited explainability and lack of transparency are the most heavily debated as they create important challenges in ensuring data privacy, patient autonomy, and consent [12]. Wide recognition of these challenges has led to an abundance of ethics guidelines and implementation frameworks regarding the development, use, and deployment of AI by industries, academic institutions, and governmental bodies [13]. A widely cited example is the European Commission’s *Ethics Guidelines for Trustworthy AI* (hereafter referred to as EGTAI) aimed at providing a general approach to dealing with AI related risks and ethical concerns [14–16]. Initiatives specifically addressing the medical setting include the Dutch *Guideline for high-quality diagnostic and prognostic applications of AI in healthcare* [17], the European *FUTURE-AI* initiative [18], and the *Ethics and governance of artificial intelligence for health* commissioned by the World Health Organization [19].

However, embedding ethics into the development and implementation of AI in clinical practice has proven difficult [13, 20, 21]. Firstly, the highly abstract nature of most guidelines, which are often principle-based, is deemed inadequate for accurately estimating the potential impact that AI-driven technologies will have within their specific contexts of use, including existing healthcare processes and practices [20–22]. Secondly, even though patients are the primary beneficiaries of healthcare technologies, the majority of AI ethics guidelines and policy documents have excluded patients (and citizens, more broadly) from direct involvement in the development of these documents or only involved them at a later stage in the process, and, even then, usually indirectly through patient organizations [14, 17–19, 22, 23]. Previous research, however, has shown that policy priorities, such as those set out in medical research agendas, do not necessarily align with what patients deem most crucial [24]. Moreover, a lack of patient involvement deprives them of having their voice heard and undermines the principles of procedural justice [25]. Therefore, failing to incorporate patient perspectives into development and implementation of AI technologies, such as the prediction model that PROFID aims to develop, would be a major omission. This is also acknowledged outside of Europe, as the American Heart Association has recently emphasized the necessity of integrating the patient perspective into the development of AI-driven tools [26].

Qualitative research on AI in medicine initially focused on medical professionals’ views [27–31], and researchers have only recently started addressing patients’ perspectives [32–38]. Only a few studies have explicitly explored ethical questions from the perspective of patients [39–41] and none of these focused on cardiology, much less on SCD prevention. The aim of our study is therefore to empirically explore the patient perspective on the ethical use of AI in the setting of SCD prevention and ICD implantation. We used an empirical ethics approach, which aims to understands through empirical research how normativity exists within a particular context [42]. This is in contrast with approaches attempting to determine in advance what constitutes ‘good’ in a particular context. Therefore, in our study, we focus on how the contextualized experiences of cardiac patients shape their perspectives on the ethical use of AI in healthcare.

In this paper we present our findings based on in-depth interviews with patients who carry an ICD and patients at increased SCD risk who do not (yet) carry an ICD, regarding their perspectives on the ethical use of AI in the clinical setting of SCD prevention and ICD implantation. In the discussion section, we will reflect on how our findings underscore the critical necessity of integrating the patient perspective into the development and implementation of AI in medicine. Furthermore, we will show how our research, while centered on SCD prevention, transcends its specific context and discuss its implications at three different levels: clinical practice, AI ethics research, AI health policy.

## Methods

### Recruitment and participants

Participants were recruited through the Dutch ICD-patient association (Stichting ICD Dragers Nederland), the Dutch Heart Foundation (de Nederlandse Hartstichting), German Heart Foundation (Deutsche Herzstiftung), and the European Heart Network who distributed our recruitment text through their networks, websites and social media platforms. Eligible participants were adults who had either an ICD and/or a heart condition with increased risk of SCD. Participants in the study contacted the researchers themselves and were then sent an information letter by email before the interview appointment was made. We used purposive sampling to best ensure an even distribution between participants with or without ICD, as well as across gender and age groups. Before the interview, we recorded participants’ written or verbal informed consent. Furthermore, the collected data has been fully anonymized, and participants’ names were pseudonymized. An exemption from requiring ethics approval was received from the ethics committee of the Amsterdam UMC (METC AMC, The Netherlands; reference number W22_417#22.493).

### Data collection

Interviews were conducted by AK and MM, with AK conducting each interview online through a video communication platform and MM conducting some interviews in person, mostly at participants’ homes, and some online. The average duration of an interview was 60 min, with a range of between 45 and 90 min. We used semi-structured interviews to gather participants’ thoughts on ethical AI use in SCD prevention and ICD implantation. The open-ended format allowed us to identify recurring themes and understand patients’ perspectives on AI’s potential impact in the setting of SCD prevention. The interview guide (Appendix 1) was jointly developed in a workshop setting by the author group, comprising both Dutch-speaking and German-speaking researchers. The comprehensibility and effectiveness of the interview design was evaluated based on a pilot interview conducted by MB and DW. In addition, MM and MB jointly conducted the initial two interviews with Dutch participants and discussed the interview guide extensively for some final adjustments for the subsequent interviews.

**Fig. 1:**
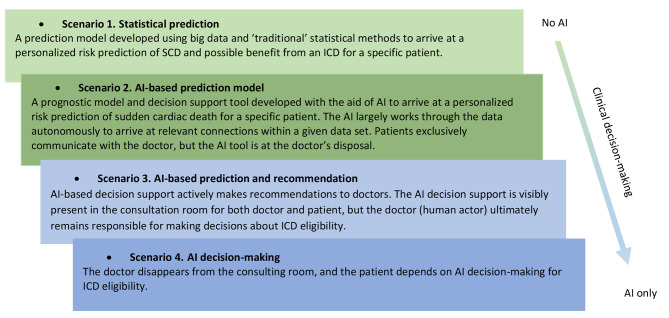
Scenario descriptions: progressive AI use in SCD prevention and ICD implantation

The interview consisted of two parts. In the first part, we invited patients to elaborate on their experience as a patient receiving and living with an ICD or having a heart condition. The second part of the interview focused on the ethical use of AI, based on four scenarios where AI played an increasingly important role in determining whether an Implantable Cardioverter Defibrillator (ICD) would be the most beneficial treatment option for a particular patient. We drew up the scenarios as follows and presented them to the participants as such (Fig. 1):

We used the Ethics Guidelines for Trustworthy AI (EGTAI) commissioned by the European Commission to facilitate participants’ ethical reflection on various scenarios based on prominent AI-related ethical themes discussed in the document [14]. We selected the EGTAI because it is widely cited and provides a relatively clear framework with seven central requirements to guarantee the development and use of ‘Trustworthy’ and ‘Human-centered’ AI-driven technologies. These requirements are: *Human agency and oversight; Technical robustness and safety; Privacy and data governance; Transparency; Diversity, non-discrimination, and fairness; Societal and environmental well-being;* and *Accountability*. Furthermore, the EGTAI is based on a normative framework consisting of the ethical principles of: *Respect for human autonomy, Prevention of harm, Fairness, and Explicability.*

During the interviews, for each scenario we invited participants to classify the seven requirements for trustworthy AI as either *‘less important,’ ‘important,’* or *‘very important’*. Each requirement was represented by a label on a computer screen that participants could move around during the interview (Appendix 2). In addition, we encouraged participants to reflect on the following topics in addition to the seven requirements from the EGTAI document: ‘*Trust’* and *‘Shared decision-making and the doctor-patient relationship*’. Each of these topics had its own label on the screen as well. By adding *‘Shared decision-making & doctor-patient relationship’*, we invited participants to specifically consider how introducing clinical AI may affect the doctor-patient relationship and the role of patient autonomy within this dynamic. Furthermore, while *‘Trust’* may not qualify as a requirement per se, the EGTAI document lacks any reflection on the concept of trust, even though trustworthiness is presented as a prominent starting point for the document. Finally, participants had the option to suggest a topic themselves, which was represented by a label with a question mark. This meant that participants had the opportunity to classify a total of ten labels per scenario according to their degree of importance. The purpose of this classification was primarily to facilitate the conversations.

### Data analysis

Audio recordings of the interviews were converted verbatim into interview transcripts in the original language spoken during the interview. This resulted in two sets of transcripts, one in German and one in Dutch. We first carefully reviewed all the interviews and made notes and memos to develop a comprehensive dataset overview. AK and MM coded both initial datasets independently in the original language of the interviews in MAXQDA 2022. We employed deductive coding, following the EGTAI requirements as per interview design, and subsequently inductive coding, whereby we engaged in qualitative content analysis [43]. Subsequently, AK and MM discussed both resulting code systems and jointly incorporated them into a shared code system (Appendix 3). Throughout this process, we took into account the potential impact of language differences by comparing quote examples for each code to ensure consistency in coding across both language datasets. The impact of language differences turned out to be minimal. The resulting code system was discussed in a coding session with MM, MB and DW, after which the revised code system was applied to the entire dataset. Additionally, one Dutch and one German native-speaking researcher not directly affiliated with the study each coded an interview. Their feedback was utilized for a review of the code system. The illustrative quotes used in this paper have been translated from Dutch and German into English in the drafting phase of this paper. Given that translation requires interpretation, the selected quotes were assessed by AK (DE) and MM (NL) for their original meaning [44]. Finally, this study’s reporting follows the Consolidated Criteria for Reporting Qualitative Study [45].

## Results

### Study sample

We interviewed seven women and eight men from the Netherlands (*n* = 15; mean age, 57; range, 30–74), and two women and seven men from Germany (*n* = 9; mean age, 62; range, 38–76). At the time of the interview, eleven participants had an ICD for a duration varying from just under a year to twelve years. Thirteen patients without an ICD had a medical condition associated with an increased risk of SCD. The participants without an ICD had inherited cardiomyopathy, ventricular arrhythmias, coronary artery disease or previous myocardial infarction. A minority of participants with an ICD received the device due to experiencing a myocardial infarction combined with decreased LVEF, per the European ICD implantation guidelines, while for others, ICD implantation resulted from, for example, the presence of a genetic cardiac anomaly (e.g., Long QT Syndrome, Phospholamban-associated cardiomyopathy [46] or reduction in LVEF due to factors such as anti-cancer drug treatment.

### Patient perspectives on the ethical use of AI for sudden cardiac death prevention

Participants interpreted the EGTAI requirements in a manner consistent with the definitions of these concepts as presented in the EGTAI (see Appendix 3 for an overview of interview codes). However, while the EGTAI primarily outlines the requirements that AI design should adhere to, participants rather considered how healthcare workers, especially doctors, would interact with AI driven technologies, and how this would affect the patient experience in clinical practice. This was evident from the way participants responded to the different scenarios we presented, in which the role of AI became increasingly important in each consecutive scenario.

Participants’ responses to the first two scenarios sharply contrasted with their reactions to the last two. Regarding the transition from the first to the second scenario, most participants saw using AI for improving risk prediction (Scenario 2) as primarily affecting the doctor’s work and professional practice, rather than the patient directly. They believed patients’ understanding of how doctors exactly do their work is already limited, and did not anticipate that using AI would result in significant changes to the patient experience. However, as the scenarios progressed towards the third scenario, and AI became actively involved in clinical decision-making, concerns arose about how the use of clinical AI would affect them as patients. Consequently, participants increasingly emphasized the importance of what they commonly referred to as the ‘human touch’ or the ‘human dimension’ in clinical practice. This sentiment became particularly clear in the fourth and final scenario, in which the doctor completely disappears from the consultation room and the patient is only left with a form of AI. Participants unanimously found this scenario unacceptable and emphasized that a human being must remain responsible for the final decision at all times. To express this sentiment, participants turned to the *Human agency and oversight* requirement. However, at that time, oversight no longer solely pertained to the AI technology, but was primarily used to emphasize the desire for human oversight over patients and their care more broadly.

When discussing the scenarios, participants’ classification (ranking from *less important* to *very important*) formed the basis for the conversation. From this emerged as the most prominent in terms of importance: *Human agency and oversight*, *Transparency*, *Shared decision-making and the doctor-patient relationship*, and *Trust* (See Appendix 4). However, as participants explained their choices, they did not sharply distinguish between the requirements, but rather drew connections between the requirements. For example, some participants viewed the mere presence of a human alongside AI as a form of transparency, and thus connected the requirement *human agency and oversight* to *transparency.* Moreover, participants extensively drew upon their experiences as cardiac patients to articulate their views on the ethical use of AI. Therefore, the results are organized into six themes that arose from the interviews rather than being structured according to the EGTAI requirements.

#### Rectify human limitations with AI

Participants saw significant potential in the use of AI in the context of SCD prevention. For instance, some participants considered potential cost savings of more effective AI-based ICD indications, and others similarly emphasized environmental benefits as ICDs are essentially chemical waste after the patient’s death. Most participants, however, emphasized the potential of technological innovation in healthcare due to perceived deficiencies in the current healthcare system, including concerns about the demand for care increasingly surpassing the availability of healthcare personnel.

In addition, several participants believed that AI could mitigate human deficiencies, like stress and fatigue, that might otherwise negatively impact doctors’ judgments. However, participants unanimously supported the idea that the final decision should always be made by the cardiologist (and the patient).

*Yes, because I mean the cardiologist who, I don’t know, who sees dozens of patients a day, I also make mistakes at work, I can imagine that at number 39, the cardiologist thinks, yeah, well. Yes, that’s how it goes, let’s be honest. If a [AI] computer is involved in the analysis or monitoring, then I think, yes, that is a good development. But I do think that the cardiologist has to make the final decision about what to do* (Interview 12).

Many participants mentioned they had noticed differing opinions among specialists. Some argued that a specialist’s personal, yet professional, opinion can be too decisive in recommending a particular treatment such as an ICD. Introducing an ‘objective’ and ‘neutral’ AI technology in the consulting room could nuance the judgment of a single specialist. The following participant explicitly related this perspective to the fact that she underwent several treatments, including getting an ICD, the necessity of which she afterward considered doubtful.

*I have also experienced a few times, both with my doctor and with others [specialists], that one doctor contradicts the other doctor. Or that I have the feeling that one doctor is lax and that sometimes I could have been spared a lot of things. And then I think, yeah, such computer intelligence… then it would be nice to have [artificial] intelligence to counter the doctor’s convictions a little bit* (Interview 5).

Most participants demonstrated a good understanding of the potential risk of biases when using large data sets and AI, in accordance with the EGTAI. Some participants suggested, however, that an AI model may also help to mitigate human biases in doctors, especially with regard to external characteristics of the patient that may negatively impact a doctor’s judgment.

*Yes, I’ll say that artificial intelligence can’t make any distinctions. A doctor can pay attention to appearance: My God, he has a [certain type of] nose, I don’t like him. That can lead to rejection based on appearance, but artificial intelligence doesn’t differentiate. It doesn’t say: He has pleasant language or speaks too loudly or softly. Therefore, these personal factors become less significant when artificial intelligence is used* (Interview 16).

#### Let the facts speak for themselves

Several participants explained that they noticed differing procedures between hospitals and different opinions between specialists, leading to confusion and uncertainty. When the following participant reported severe and persistent fatigue at the first hospital she visited, her concerns were dismissed as insignificant. This left her insecure, while her complaints continued. In the hospital where she was finally treated, after repeated complaints, it was discovered that she had a genetic abnormality. She believed that AI-driven clinical decision-making, based on hard data, could boost patient confidence in the doctor and the patient’s own assertion.

*In the first period, arrhythmias were showing, and my ejection fraction was under forty. I had quite a bit of fatigue, but they [at the first hospital] thought it didn’t fit the results. While at the other hospital, the idea was that it did fit. And at the first hospital, they initially said it was probably between my ears. Those are unpleasant things to hear. So, it varies by cardiologist. (…) And yes, it depends, but I would be able to let it go more if you have the doctor’s thoughts, but in addition to the data* (Interview 7).

The solution for the problem of this participant was found to be related to the use of beta-blocker drugs. These drugs reduce SCD risk, but may have a fatigue-inducing effect in some patients, including this participant. She was taken off the medication and therefore needed another treatment to prevent SCD, namely an ICD. She was involved in these decisions, but it was difficult for her to fully comprehend the consequences of what she, in consultation with the cardiologist, would decide. According to her, having a data-driven decision support system next to the cardiologist would potentially have given her more confidence in making a decision.

*I had dangerous arrhythmias and my ejection fraction was, I think, 42%. So that was not dramatically low. Should I have gotten an ICD then, on paper, maybe not. But then I would have had to take medication, which would have prevented me from functioning here, in the family.*

[…]

*But I think that maybe if I had had a computer there and […] it had immediately become clear: okay, the ICD is really the only best solution for you. Then I would not have had to doubt whether to do it or not. It would have been like, okay, it’s not solely the doctor’s choice, but based on the data, it is the best choice. The decision would have been even more straightforward for me. But my say should still be important* (Interview 7).

#### Second opinion or not?

While some participants believed AI decision support could serve as a second opinion, others disagreed strongly. The next participant discussed why he thinks that AI decision support should not be qualified as a second opinion, and goes on to describe the extent to which the doctor should be able to rely on AI. Ultimately, only a second human doctor would be able to bear the moral responsibility a second opinion holds.

*No, absolutely not. A second opinion is a-, Then there should be somebody, another scientist or a doctor, you name it, who also looks at that data, and that they have the same opinion about-Do they have the same idea about that data? That’s a second opinion. But a doctor who says to me or somebody says: listen, your situation is like this, and from the technology, let me use that word for a moment, from the technology and data, we think you should get an ICD, or whatever, then I don’t think that’s a second opinion. Then that’s just data – Precisely the same as what the cardiologist gets from a heart monitor or a blood test. Yes, it’s more of an addition than a second opinion* (Interview 8).

In addition, several participants indicated they would be more understanding of a medical error by a human doctor compared to an error by an AI-driven technology.

*I imagine if I’m dealing with a human being I can also think yes, that can happen. That can happen. (…) The human aspect has a very big role. I personally think I will be more rigorous. If there is only artificial intelligence-If I am dealing with that. (…) Yes, still then-And I find that very strange that I say that actually, because then I expect more from the AI than from the cardiologist* (Interview 11).

#### Explainability, transparency and trust

Participants agreed that it is important for clinicians to assess the AI system for reliability and that it should be mostly explainable to them. However, expecting the same from patients would be a bridge too far. One participant noted that even now, patients can access blood test results and see how they compare to the normal values which does not mean, however, that you, as a patient, are able to explain the significance (or cause) of a particular below-normal level. The same goes for AI; this participant argued: “Smart people have been working on that, and I have to have faith in that. Then I should not want to understand all that because it would entail an entire study in itself” (Interview 2).

Others stated that they would have complete confidence in the system if it were a human doctor who communicated AI results, simply because of the doctor’s presence. When asked whether this task could not be performed, for example, by a specialized nurse, participants unanimously answered they did not think so. The primary reason for this was attributed to doctors’ expertise. Doctors were considered especially equipped to empathize with patients’ situations from a medical standpoint, and could therefore serve as sparring partners to interpret the relevance of AI outcomes with the patient. However, if the doctor were completely out of the picture, several patients would want to, literally, question the AI decision tool on its reasoning: “So that it is clear and understandable to me as a patient how that system comes to its conclusions [and] get some kind of instruction or explanation of how the system did it” (interview 3). Above all, patients wanted to be presented with a track record of past performance and an overview of the number of patients who came before them.

Several participants expressed that they expected that the sheer amount of data associated with AI-driven technologies would hinder transparency. They emphasized the ongoing importance of trust and confidence in doctors.

*So much information is also making it more complicated to be transparent. It already sometimes gets very technical, and in that regard, the cardiologist is the expert. And I, as a patient, interpret it differently anyways. And that’s why trust is important. And I also have to be able to put it out there if I have any doubts. And in that respect, whatever the case, I desire to have a human to assist me whenever I require help* (Interview 11).

The following participant emphasized that when in a vulnerable position, you should be able to trust the care provided by the hospital and believe that the hospital has your best interests at heart. For her, the same would apply if a hospital decided to switch to the use of AI. She emphasized this attitude using the example of when she initially received an ICD.

*It’s quite a hassle because it’s very painful to have such a wire, everything goes under local anesthesia, and that wire is not very pleasant. Someone representing the manufacturer is also present during the implantation. They come up to you and show you the device and the wire and tell you a few things (…) but I thought: if that is the right one, just put it in. I’m sure the doctor knows what to do, I’m just a layperson, and you must trust that* (Interview 1).

#### ‘The human touch’ in clinical practice

The majority of participants emphasized the importance of what they described as the *‘human touch’* in clinical practice: compassionate and dignified care that considers not only the physical but also the mental well-being of patients. However, participants also indicated this need is not always met in current clinical practice. Many participants believed doctors prioritize clinical guidelines excessively and appear constrained by time. One participant half-jokingly stated that if it got any worse, he would “rather have a 60-minute conversation with a computer than sit in front of [a] person”, meaning the physician (Interview 17). Participants primarily pointed to the ever-increasing workload in healthcare as a cause for this. Participants feared that the introduction of AI would not take away from this dynamic, but rather only further dehumanize care.

One of the participants indicated that what he felt was missing in the EGTAI requirements was something that reflected “the emotional”. This participant explained that when he initially received an ICD, his cardiologist interpreted the subjective experience of the participant by stating: “now that you have an ICD, you don’t have to worry anymore” (interview 3). This statement, however, did not reflect how the participant was feeling at the time. In fact, he felt like his state of mind was being ignored altogether. Ultimately he requested another cardiologist:

*That was part of the reason why I changed my cardiologist, because I am now in the process of only getting a periodic check-up, once a year they do an ECG and then you have to go to the cardiologist again. I asked for another cardiologist and I also said: ‘not because I don’t trust her factual judgment, but because I don’t have that emotional rapport with her and that is also important for me. Then it was also indicated right away: ‘you should absolutely indicate that too if you don’t have that, that is important’* (Interview 3).

Just as the patient above did not question the factual judgment of his original cardiologist, most participants did not expect that using AI would necessarily lead to significantly different diagnostic and treatment outcomes either, and in that respect, held similar expectations for both AI and doctors. However, many participants were concerned that an AI-driven decision would reduce the patient to a disease or a set of somatic symptoms, thus compromising human integrity and respect for one’s individuality:

*Well, I think it does something to your individuality because then you’re just patient 326 out of the system and this is the most convenient thing to do. And I think that, anyway at least for me, that for a lot of people with a chronic illness it is a challenge to not continuously having to feel like a patient. That you also have an individuality, you’re not your illness, but that it is just a part of who you are. And sometimes when I´ve been in the hospital for example, I always get a little, well not really down from that, but then it always becomes even more painfully clear to me that I´m a patient so to speak. And I can imagine with such a [AI] program I would experience that even more. Then I think, I am just one of so many. Then it’s really just ones, pluses and minuses, and then something comes out of that and that’s for me. But that is not true at all, because I am Amber, so I do need my own specific course of action* (Interview 9).

This participant proceeded to argue that, even when the course of treatment is to a very large extent beyond the patient’s control and understanding, efforts to make the patient feel valued can still encourage patient autonomy.

*I would then want to know how and why that system came up with that and what information underlies that. I also think it would become clear that you are missing the human touch or something. Of course, you want to make choices based on data and experience. But I’m also just a patient, a human being, and that should also have an impact. Even though that may not always be true in medical practice, but it’s still nice if you think it is. Because then you feel that you yourself as a patient matter or something, and that that also factors into the decision* (Interview 9).

#### Personal context, personalized care

Although the premise of using AI in SCD prevention is that it should lead to more personalized treatment, many participants expressed skepticism about whether this would actually occur. While AI might statistically entail significant improvements, statistics remain probabilistic, and there might still be a risk they say very little about the individual patient. In contrast to an earlier instance where a participant emphasized trust in the doctor and identified as a layperson, the following interview excerpt highlights the importance of involving the patient in deciding whether an ICD is indeed the right solution.

*Of course, it’s also very efficient, this is patient A and that is patient B, but then you still get distinctions, and it’s not so personal after all. And that’s why I think shared decision-making is very important. And shared decision-making is about the patient seen in the context of the patient and their environment, not just the patient sitting in the consultation room at that moment (Interview 11)*.

One participant emphasized that while technology can be beneficial in certain cases, its use should not overshadow the primary focus of medicine: providing treatment and care tailored to the patient’s unique circumstances and needs. She stressed that this is something that cannot be done by a computer on behalf of healthcare practitioners. She expressed particular concern about how healthcare workers perceive and use these technologies, both in terms of their expectations and actual implementation.

*Well, if you look at the personnel shortages of doctors and nurses, I do believe the computer could play a more prominent role. But I’ve noticed that nurses in the hospital are often more concerned with the computer than with you, the patient, while they could also come over and ask me. I know what medicines I am taking. But no, all of that has to go through the computer. And people think that saves time, but I seriously wonder about that. And maybe there are fewer errors percentage-wise overall (…) But I hope that it doesn’t end up being that way, that [AI’s] role becomes so big that the human, that’s what I just meant by human, that the doctor and the nurse forget that they’re the ones who have to do the work, and not that computer. [The computer] can only look at the data you put in, but not everything beyond it. And that is just as important, and sometimes even more important.* (Interview 6).

## Discussion

In this study, we conducted semi-structured interviews to explore the perspectives of patients at increased risk of sudden cardiac death (SCD) regarding the ethical use of AI in the context of SCD prevention and implantable cardioverter-defibrillator (ICD) implantation, with a particular focus on clinical decision-making. Participants viewed AI-driven prediction and decision-making as more objective than that of doctors. Yet participants believed it is essential for the doctor to assess the AI application for reliability and effectiveness; this is not something that patients can or should do themselves. In addition, they saw a role for the doctor as a sparring partner to see if and how an intervention fits into the lives of patients. Hereafter, we discuss how our findings contribute to the literature on the ethics of medical AI and should be used to complement existing guidelines and to guide further research and clinical practice.

### Shared decision-making and (Un)explainable AI

In both the academic discourse [12] and contemporary AI ethics guidelines, such as the EGTAI, the increasing complexity of certain AI systems emerges as a prominent concern, especially within the medical context. Some argue that the use of these ‘black-box’ systems could diminish physicians’ comprehension of medical processes and challenge their epistemic authority, potentially conflicting with the fundamental principles of patient-centered medicine and thereby ultimately eroding patient trust [47–49]. This concern becomes particularly relevant in the practices of shared decision-making (SDM) and ensuring informed consent, both firmly grounded in the principle of respecting patient autonomy. SDM involves a dynamic exchange between doctor and patient, wherein the patient receives sufficient and comprehensive information to make informed, rational decisions aligned with their values and goals [50, 51]. To facilitate SDM, many scholars have advocated the adoption of explainable AI (often referred to as XAI) in medicine [12, 52–54].

However, even if experts come to agree on a general standard for explainable AI, there will still be a challenge in effectively communicating AI-driven recommendations to patients, which would possibly even necessitate efforts to improve patient literacy to ensure shared decision-making and informed consent [55]. Even so, we question the feasibility and acceptability of such efforts, especially in light of our results, which suggest that this could potentially excessively burden patients with a responsibility that they feel should remain with the doctor. Moreover, some authors have observed that healthcare access and health literacy are already unevenly distributed in societies, and expecting patients to take on a more proactive role may exacerbate existing health inequalities [56]. Furthermore, others have contended that medicine, in its own right, can be viewed as a black box [57]. Just as many of our participants expressed, also without AI, patients often find themselves in a position where they have to rely on medications and treatments that they may not entirely comprehend; a situation that, to some extent, also applies to the specialist responsible for their care [57]. Our finding that patients above all want to know how certain recommendations, regardless of how exactly these technically came about, may affect their daily lives, is not unique to the context of clinical AI. In current clinical practice, bridging the gap between patient values and physicians’ evidence-based opinions is already widely regarded as challenging [58, 59]. Also, within evidence-based medicine, which necessitates the integration of patient values with the best clinical evidence, the role of values lags behind [60]. There seems no reason to believe that a perfectly explainable, and perhaps even widely deemed trustworthy, AI will resolve these issues; in fact, it might even divert attention from the doctor’s crucial task of effectively discussing the relevance of outcomes with the patient [61].

### The importance of a human doctor

A significant portion of our results centers on the question of whether AI could replace human doctors and what potential trade-offs might be involved in the transition towards AI-driven tools. Patient perspectives on the perceived advantages and disadvantages of AI often shared common elements, such as the ‘human dimension’. This term was explicitly invoked by participants to address human qualities like empathy but was also implicitly used to refer to human limitations, such as biases, fatigue, and fallibility, against which participants contrasted certain favorable attributes of AI.

Our results demonstrate that although participants attribute a high degree of objectivity to AI when it comes to data-based outcomes, they do not think that an AI is capable of bearing the moral responsibility medical decision-making entails. This idea is further highlighted by the fact that some of our participants expressed that they would be more forgiving of human error compared to AI errors. Possibly this points to a phenomenon commonly referred to as ‘algorithm aversion’, where people tend to assess human actions more favorably compared to an algorithm performing the same action [62]. Our participants believe, however, that only a doctor is capable of effectively assessing a patient’s specific situation and values, and, therefore, the doctor should not disappear from the consultation room.

Consistent with previous studies, our participants emphasized the importance of relational aspects in healthcare and the critical role of doctors in maintaining human dignity through empathy and understanding [34–36, 38]. For example, even when decision options seem limited, doctors are still presumed to be capable of fostering a sense of self and autonomy in patients by treating them with respect and integrity. However, this notion is not limited to mere kindness in the medical profession, as empathy holds significant clinical relevance. Previous research suggests that defining empathy should extend beyond an individual’s ability and emphasize it as an ongoing reciprocal dynamic between doctor and patient [63]. Patients are, for example, known to be selective in what information they disclose based on their trust in the empathizer’s intentions [64]. Therefore, it is particularly relevant to consider the potential impact of AI on these dynamics within the doctor-patient relationship.

In their 2023 study, Ayers and colleagues [65] found that responses from a chatbot (ChatGPT) to randomly selected patient questions on an internet forum were preferred by patients over those from physicians, receiving significantly higher ratings for both quality and empathy. Others, however, have argued that AI, such as an AI chatbot, may very well demonstrate what they call ‘emotional empathy’; recognizing the emotional states of patients, but lacking authentic intentions to help, for instance. Only a human doctor, on the other hand, is capable of what they call ‘motivational empathy’ [64]. Motivational empathy recognizes the fact that demonstrating authentic empathy is taxing for the empathizer, as it requires a willingness to invest effort and time in understanding and acknowledging the recipient [64]. In line with this, our results point to the fact that the doctor’s role to help patients navigate challenging situations, such as receiving an ICD, should not be underestimated, and is indeed tied to the doctor’s perceived positive and genuine empathetic intentions towards the patient. It is not without reason, for example, that ‘breaking bad news’ effectively is considered a crucial skill in many critical clinical settings. The way this is done can significantly impact the subsequent course of care and the steps patients’ themselves are willing to take [66].

Previous studies have further explored various challenges associated with relying on AI for medical decision-making and the moral judgments that are woven into it [67, 68]. These authors pointed to the fact that AI systems, as it currently stands, entirely rely on past data and are thus unable to predict entirely new human behaviors or handle variables like chance. Even when these factors are included into AI analysis and medical decision recommendations, elements such as patient preferences, would need to be accounted for by a certain statistical weight. Determining a predefined cut-off point beyond which a patient’s value, for example, can no longer be justified from a predefined biomedical perspective, becomes necessary [67]. The question is whether such an application of AI would be desirable, as this might come at the expense of the more flexible reflective capacity of the human moral agent. What patients find most important, varies, and what might be a perfectly logical consideration for one patient could seem completely irrational to someone else. Moreover, the prospect of clinical decision-making solely based on probability calculations was one of the things our participants were particularly concerned about. Our results highlight the importance of the human doctor as sparring partner to interpret the relevance of AI recommendations with the patient, on an equal moral footing, to ensure that practices of shared decision-making occur in an effective, and valuable manner. Therefore, we argue that patients should not only retain the right to a human (medical) decision, an idea that others have explored [69], but also retain the right to a human doctor.

### Implications for practice and further study

In what follows, we discuss the implications of our research at three different levels: clinical practice, AI ethics research and AI health policy.

First, our results have implications for clinical practice. The use case we outlined in this study concerns decision-making around ICD implementation, a case which is particularly timely due to the lack of good evidence for clinical guidelines and pertinent within policy contexts. Studies supporting current guidelines are often more than 20 years old (the PROFID project aims to update these with new evidence). In the Netherlands, in particular, there has been much attention for this topic. The Dutch Healthcare Institute (Zorginstituut Nederland) recently sounded the alarm about the state of ICD implantation [9]. While annually more than 6,000 Dutch patients receive an ICD, recent research indicates that in a significant proportion of these patients, the placement of an ICD does not effectively prevent mortality, and there is a considerable risk of complications. Moreover, patients frequently receive insufficient or incorrect information about potential complications associated with ICD implantation, hindering shared decision-making and patient autonomy [9]. While information provision varies among institutions, and not all Dutch cardiologists endorse the Institute’s conclusions, our findings firmly underscore the Dutch Healthcare Institute’s call for more personalized and patient-centered care in the context of ICD implantation. Given these findings, we are planning an ethnographic observation study on ICD implantation in the Netherlands to better understand how patient-centered innovation can be accomplished within this particular context and beyond.

Secondly, we underscore the implications of our research for AI ethics. We suggest that a deeper understanding of AI’s possible impact in healthcare could be achieved through a comprehensive, context-specific, and empirical approach. Social science scholars, especially those in Science and Technology Studies, have long emphasized that technology’s effects are not always fixed or even predictable [70]. Similarly, participants in our study emphasized that they expect the impact of AI will depend on the specific approach taken by healthcare practitioners, especially doctors, in their use of such technologies. To account for the wide variety of contexts of use and consequential outcomes, others have highlighted the importance of ethnographic and observational methods in AI research [71], but the uptake of these methods in AI ethics has so far been minimal. However, we believe that researchers in empirical ethics, in particular, are well-suited to undertake this task. As mentioned above, we will actively participate in this as well. In the context of care practices, empirical ethics describes the goals, values, norms, and undesired outcomes that caregivers and patients pursue and aim to avoid [42]. Thus, beyond the well-founded principled concerns surrounding complex AI systems, such as those outlined by the EGTAI, there exists a nuanced reality regarding how patients’ values, such as autonomy, manifest in the everyday, often mundane, ethics of clinical practice. Ethnographic research is essential for a detailed understanding of clinical practices, necessary to guide the transformative potential of technological innovations in health care, such as medical AI [42].

Third, our results hold significant implications for AI health policy. We believe that our findings provide reason for advocating the right to a human doctor, thus ensuring meaningful patient involvement in the processes of diagnosis, treatment, and care. Our position is rooted in the recognition that healthcare decisions can involve complexities beyond the capabilities of AI [67]. We argue that these complexities include weighing patient preferences against the best available clinical evidence and this necessitates a human touch. Further normative research is required to determine what such a right to a human doctor would entail and, subsequently, whether this would also be legally feasible [69]. We do believe this should involve input from various stakeholders, with a particular emphasis on giving the patient’s voice a central role in this discussion; ultimately, it is their well-being and health that is at stake. Moreover, it is important to recognize that this also applies to other healthcare practitioners, such as nurses, who play a significant role in patient care. The use of AI will inevitably impact them as well.

### Strengths and limitations

Our study is among the first to focus on patients’ perspectives on the ethical use of AI in a particular clinical setting, particularly with regard to prevention. However, our study is not without limitations. While recruiting participants through patient organizations was effective, it also resulted in a relatively homogenous study sample with regard to ethnic, cultural and socio-economic characteristics. Further, the majority of candidates found our recruitment call through various digital platforms. Consequently, individuals with lower levels of digital literacy may not have been included while they may very well have different concerns and needs related to AI and digitalization in healthcare. Additionally, participants often found it challenging to clearly distinguish between some of the scenarios presented. This might have been due to the fact that participants were aware in advance that AI would play a central role in the interviews. As a result, participants promptly shared their opinions on the use of AI in healthcare, sometimes regardless of the specific scenario presented. Our results might also be biased toward those who had more affinity with the topic of AI. To mitigate these limitations, we included researchers from different disciplines to approach the data with as open a view as possible. Additionally, the large number of eligible patients interested in participating in our research allowed us to apply a high degree of purposive sampling, considering the distribution of cardiac patients with and without ICD, as well as gender and age. Further research is needed to assess how such factors play a role in patients’ perspectives regarding ethical AI. Finally, we have not managed to include all themes from the interviews in the paper and have focused on the points that patients found most important instead. On the other hand, this has enabled us to describe the included themes in greater depth.

## Conclusion

The top-down approach, upon which most AI ethics guidelines are currently based, provides a valuable starting point for navigating the potential risks posed by using AI in clinical settings. However, it only offers a limited perspective in which the patient’s voice is still missing. By addressing this shortcoming through interviews with patients in the context of SCD prevention, our results are relevant on multiple levels. First, they demonstrate patients’ desire for more personalized and patient-centered care in the context of ICD implantation and confirm the increasingly loud call in research and policy to achieve this. Secondly, our results demonstrate that patients are most concerned with the potential loss of various forms of the ‘human touch’ in healthcare, which they believe is already inadequately recognized in current clinical practice. Participants attribute to doctors the responsibility of evaluating AI recommendations for clinical relevance and aligning them with patients’ individual contexts and values, in consultation with the patient. AI is insufficiently capable of meeting this requirement, and therefore, we suggest that our findings warrant further normative research into the ‘right to a human doctor’. Finally, policy guidelines for integrating AI into clinical practice should include not only principle-based ethics but encompass the ethical considerations involved in everyday clinical practices as well. We suggest that an empirical ethics approach based on ethnographic research is exceptionally suitable to guide the way forward and ensure context-specific, patient-centered clinical practice and AI integration.

### Electronic supplementary material

Below is the link to the electronic supplementary material.

Supplementary Material 1

Supplementary Material 2

Supplementary Material 3

Supplementary Material 4

## Data Availability

The interview guide (Appendices 1 and 2) and a thematic overview of the interview results (Appendices 3 and 4) are available as additional files. Interview transcripts are, as agreed with interview participants, not publicly available due to privacy concerns. Researchers interested in more details about the interview data, may contact the corresponding author.

## Footnotes

1 https://profid-project.eu/project/.

2 At the start of the consortium in 2020, AI was used to develop an initial risk prediction model to be tested in a parallel non-inferiority and superiority trial. However, AI’s prominence in the project has diminished over time as the model did not suffice as basis for the planned trials. The current PROFID strategy is centered on a non-inferiority trial, The PROFID EHRA trial, which compares optimal medical therapy (OMT) versus ICD implantation plus OMT in post-MI patients with LVEF ≤ 35% who would receive an ICD according to current clinical guidelines. The PROFID EHRA trial aims to determine the potential benefit or harm of routine prophylactic ICD implantation for primary prevention of SCD in the setting of contemporary OMT in post-MI patients with reduced LVEF ≤ 35%. AI is still included in sub-studies within the project aimed at exploring the potential of novel and promising risk markers for personalized risk prediction of SCD, including using AI-based analysis of twelve-lead ECGs.

## Abbreviations

AI: Artificial Intelligence
AV Block: Atrioventricular block
ECG: Electrocardiogram
EGTAI: Ethics guidelines for trustworthy AI
ICD: Implantable cardioverter defibrillator
LVEF: Left ventricular ejection fraction
PROFID: PRevention Of sudden cardiac death aFter myocardial Infarction by Defibrillator implantation
PROFID EHRA: PRevention Of sudden cardiac death aFter myocardial Infarction by Defibrillator implantation European Heart Rhythm Association
SCD: Sudden cardiac death
