# Correction: Ethical use of artificial intelligence to prevent sudden cardiac death: an interview study of patient perspectives

**DOI:** 10.1186/s12910-024-01047-7

**Published:** 2024-05-14

**Authors:** Menno T. Maris, Ayca Koçar, Dick L. Willems, Jeannette Pols, Hanno L. Tan, Georg L. Lindinger, Marieke A.R. Bak

**Affiliations:** 1 Department of Ethics, Law and Humanities, Amsterdam UMC, University of Amsterdam, Amsterdam, The Netherlands; 2 Institute for Healthcare Management and Health Sciences, University of Bayreuth, Bayreuth, Germany; 3 Department of Anthropology, University of Amsterdam, Amsterdam, The Netherlands; 4 Department of Clinical and Experimental Cardiology, Amsterdam UMC, University of Amsterdam, Amsterdam, The Netherlands; 5 Netherlands Heart Institute, Utrecht, The Netherlands; 6 Institute of History and Ethics in Medicine, TUM School of Medicine, Technical University of Munich, Munich, Germany

BMC Medical Ethics (2024) 25:42

10.1186/s12910-024-01042-y

In this article [1], the Box 1 was inadvertently structured and published as Fig. 1. The actual Fig. 1 was published as Fig. 2. This has now been corrected. The text citations for Box 1 and Fig 1 in the article are revised. The corrected Box 1 is given below: Box 1 Clinical context of the PROFID project: Sudden Cardiac Death prevention through ICD-implantation The original article has been corrected.

**Box 1:** Clinical context of the PROFID project: Sudden Cardiac Death prevention through ICD-implantation

Sudden cardiac death (SCD) is a significant cause of mortality, accounting for 20% of all deaths in high-income societies [4]. For individuals at increased risk of SCD, the implantable cardioverter defibrillator (ICD) has proven to be an effective intervention, also when implanted prophylactically (primary prevention, i.e., before an SCD-causing cardiac arrhythmia has occurred) [5]. Current European guidelines for ICD implantation for primary prevention of SCD in patients who have previously experienced a myocardial infarction are solely based on the presence of a reduced (< 35%) left ventricular ejection fraction (LVEF) [6]. However, using these guidelines, actual appropriate ICD shocks are only delivered in a small proportion of patients, while SCD mostly occurs in patients who do not meet this eligibility criterion for ICD placement [5]. In other words, under current guidelines there is a discrepancy between patients who receive an ICD and patients who would benefit most from it. Moreover, ICD implantation involves inherent risks for the patient. One in ten ICD patients experiences at least one serious (sometimes potentially life-threatening) complication following implantation, most commonly related to the ICD-leads. These complications can include local and systemic infections and cardiac perforation [7].
Moreover, the fear of receiving a shock and the actual occurrence of both appropriate and inappropriate ICD shocks can lead to prolonged psychological distress [8]. Clearly, there is a need for better prediction of SCD risk in these patients, on which improved guidelines may be based.
