# Supplementary material for: Ethical use of artificial intelligence to prevent sudden cardiac death: an interview study of patient perspectives
Source: BMC Med Ethics. 2024 Apr 4;25:42. doi: 10.1186/s12910-024-01042-y (PMC10996273; doi:10.1186/s12910-024-01042-y)
Supplement: Supplementary file 1 — Supplementary Material 1 [file 12910_2024_1042_MOESM1_ESM.docx]

**Appendix 1.**

**Interview guide**

**Introduction** – entry, signing consent agreement, start of recording. (~ 5 min.).

PROFID is about finding the best possible way to determine which patients would benefit most from the implantation of an internal defibrillator (ICD) to reduce the risk of sudden cardiac death. It primarily focuses on prevention and individuals who have not experienced a cardiac arrest. In patients who have previously had a heart attack, persistent weakness in heart function is considered a high risk for sudden cardiac death. This is indicated by the ‘ejection fraction’. The ejection fraction represents how much blood your left ventricle pumps out per heartbeat to the rest of the heart. This percentage typically hovers around 60%. However, if it falls below 35%, the patient is considered to have an elevated risk of sudden cardiac death according to current insights. Currently, this percentage in combination with the occurrence of a heart attack, or myocardial infarction, is the primary indication for advising an ICD. The goal of PROFID is to develop a risk model based on extensive patient data that takes into account various other factors in addition to ejection fraction. This is to ensure that those receiving an ICD truly benefit the most, as previously mentioned, and that individuals who would have received an ICD according to current guidelines may no longer require one, thus saving unnecessary risks associated with getting and living with an ICD.

**Part 1.** (~ 10 min.)

- Discuss the background of the interviewee, including their experience with ICD implantation and any heart conditions or health issues.
- What led you to participate in this interview?

**Part 2.** (~40 minutes)

2.1 (~ 5 min.) Explanation of interview structure & procedure – 4 scenarios in which the role of artificial intelligence increasingly plays a part. On the screen, you will see several concepts that the EC thinks need to be met to considered in order to guarantee trustworthy AI. For each scenario, I would like you to categorize the requirements based on your perspective and the level of importance you would attribute to each of the requirements:

- Privacy and data governance – Is your data handled confidentially?
- Trust
- Accountability – the extent to which all the above points are met, the responsibility and accountability for AI systems should be ensured.
- Human agency and oversight – the ability for people to act autonomously regarding AI and understand and control AI systems.
- Diversity and non-discrimination – AI should be used for the sake of equality (e.g., in medical treatment) and equal access to AI systems.
- Transparency – the functioning and outcomes of AI systems should be explainable. You can also think of a trade-off between accuracy and explainability.
- Societal and environmental well-being - The sustainability and ecological responsibility of AI systems should be encouraged, as well as social consequences, society, and democracy.
- Shared decision-making
- Technical robustness and safety - a preventive approach to risks and ensuring they behave reliably and as intended.
- Question mark – you can use this for a concept or topic you think is still missing.

2.2 – Discussion of scenarios (~35 minutes).

- **Scenario 1.** – Statistical prediction: a prediction model using big data and ‘traditional’ statistical methods to arrive at a personalized risk prediction of SCD and possible benefit from an ICD for a specific patient.
- **Scenario 2.** – AI-based prediction model: A prognostic model and decision support tool developed with the aid of AI to arrive at a personalized risk prediction of sudden cardiac death for a specific patient. The AI largely works through data autonomously to arrive at relevant connections within a given data set. Patients exclusively communicate with the doctor, but the AI tool is a the doctor’s disposal.
- **Scenario 3.** – AI-based prediction and recommendation: AI AI-based decision support actively provides recommendations to doctors. The AI decision support is visibly present in the consultation room for both doctor and patient, but the doctor (human actor) ultimately remains responsible for making decisions about ICD eligibility.
- **Scenario 4.** - AI-based decision-making about ICD placement, the doctor disappears from the scene (the AI system decides on the best treatment).

2.3 (~ 5 min)

- Now, we've discussed four different models. When you think about the development of technology, what do you expect from the development and use of technology in medicine? What aspects must absolutely be considered?

**Ending**

- Do you have any final comments before we conclude the interview? Is there anything you believe has gone unaddressed?
- How did you experience the interview? Do you have any points for improvement?
