# Supplementary figures and images for: Ethical use of artificial intelligence to prevent sudden cardiac death: an interview study of patient perspectives
Source: BMC Med Ethics. 2024 Apr 4;25:42. doi: 10.1186/s12910-024-01042-y (PMC10996273; doi:10.1186/s12910-024-01042-y)

**Appendix 2.**


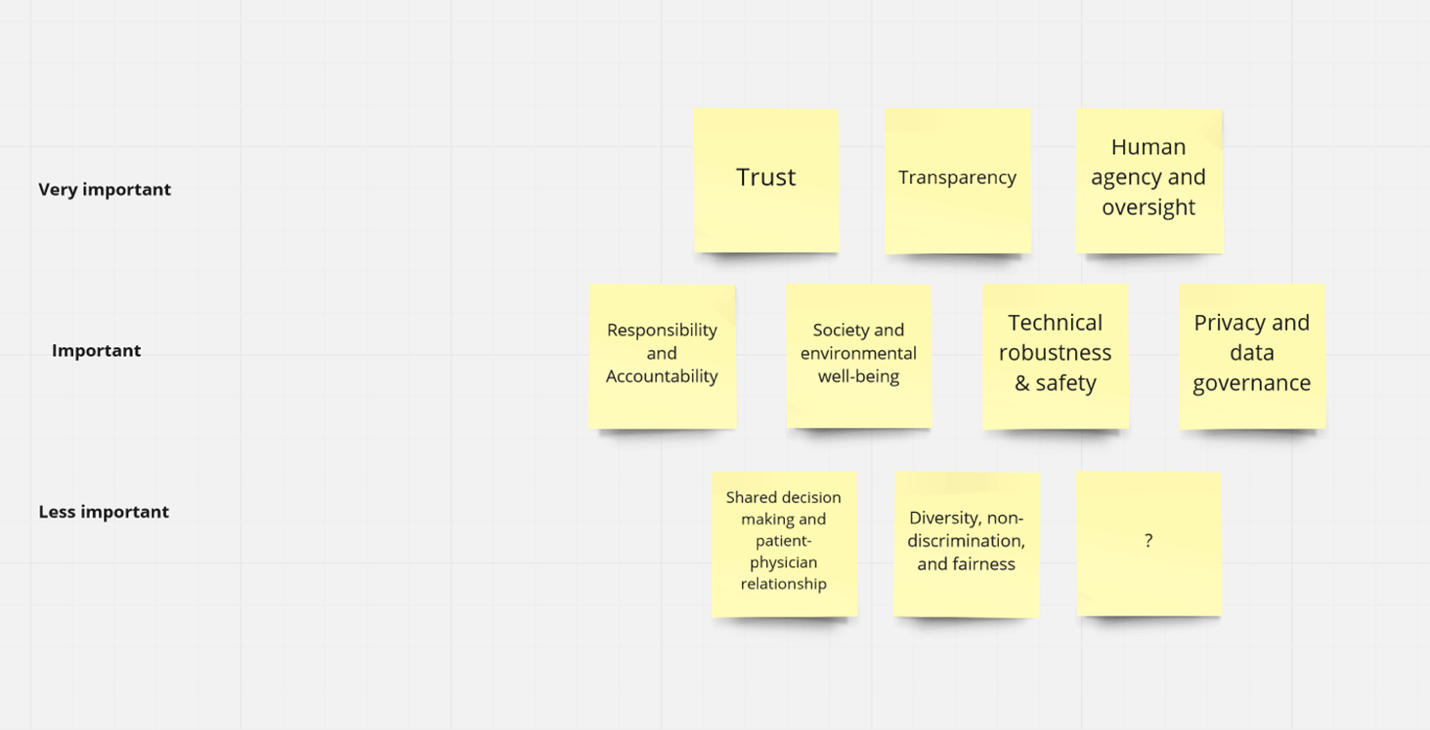
**Example screenshot of interview supplement: label classification.**

Supplement: Supplementary file 2 — Supplementary Material 2 [file 12910_2024_1042_MOESM2_ESM.docx]
