# Supplementary material for: Ethical use of artificial intelligence to prevent sudden cardiac death: an interview study of patient perspectives
Source: BMC Med Ethics. 2024 Apr 4;25:42. doi: 10.1186/s12910-024-01042-y (PMC10996273; doi:10.1186/s12910-024-01042-y)
Supplement: Supplementary file 3 — Supplementary Material 3 [file 12910_2024_1042_MOESM3_ESM.docx]

**Appendix 3.**

**Overview of interview codes**

| **Requirements** | **EGTAI definition** | **Underlying ethical principle (as proposed by EGTAI)** | **Overview of interview codes** |
| --- | --- | --- | --- |
| Human oversight and agency | Ensure users can make informed decisions in accordance with their intentions and objectives and have access to information and resources that allow them to understand and interact with AI-these systems. Whenever possible, users should be able to assess or question the system in an informed manner. | Respect for human autonomy | - Human to ensure relevance AI recommendation for patient - Human oversight as doctor responsible for treatment and care - AI as objective actor (data, doesn’t differentiate on personal traits, doesn’t fatigue) - AI will ultimately outperform human (data analysis and diagnose) - Trust through human presence (oversight) in consultation room - AI as 2^nd^ opinion - As not suitable as 2^nd^ opinion. - Continuous monitoring and assessment on data (ML) |
| Technical robustness and safety | Proactively prevent data disclosure and unwanted operational changes to ensure human integrity. Fallback plans are could involve switching to rules-based procedures or human intervention. Reproducibility and replicability are also deemed essential for a stable and reliable AI system. | Prevention of harm | - Calamities (e.g., electric fall-out) - AI – should stick to what it was designed for - Technology as value free |
| Privacy and data governance | Document and monitor data processing steps, proactively address biases in datasets prior to training AI systems, and establish clear data protocols for authorized access including restricting data availability solely to designated personnel. | Prevention of harm | - Personal and public health takes priority over privacy - Data access essential for thorough analysis - Privacy complicating factor; burdensome for patient - Thorough anonymization is essential. - Absence of human increases privacy concerns (e.g., signing agreements in absence of human) - Hacking risk; malicious intent - Medical data particularly sensitive - Technology could track behavior (e.g., ads) - Threat of commercial use of data |
| Transparency | The EGTAI document promotes transparent AI systems that are comprehensible to humans. It highlights the traceability of processes, trade-offs between explainability and accuracy, and considers the impact on human lives in decision-making. Users should be aware of interacting with AI applications and have the choice to communicate with humans. | Principle of explicability | - Gaining trust through transparency - Presence doctor already a form of transparency (strong preference over e.g., presence nurses) - Transparency as to the point communication (don’t beat around the bush – diagnosis/treatment) - Desire for AI operation history. - Desire for interaction with AI (absence human) - Disclosing AI is used important - AI is a black-box - AI should be comprehensible explainable for doctor - Desire for explanation understandable for patient - Patient unqualified / incapable of understanding outcomes |
| Diversity, non-discrimination and fairness | Inclusive design practices and inclusive hiring should ensure equal access and treatment, combating discrimination and stigmatization. Prejudice and discriminatory biases should be eliminated during data collection and throughout the AI system's life cycle. | Principle of fairness | - Accessibility (e.g., language) - Good data representation all patient groups necessary - AI more objective (value free) - Women underrepresented in cardiac care data - Statistics based decision-making requires some form of discrimination – cut-off point |
| Societal and environmental well-being | The impact of AI systems on society, sentient beings, and the environment should be considered. This applies to the impact of resource and energy consumption as well as the implications of AI on various aspects of daily life, social interactions, individual well-being, institutions, democracy, and political decision-making. | Principle of fairness; Prevention of harm | - Tech innovation over environmental concerns - ICD selection criteria AI – sustainability – reduce chemical waste - Treatment vs quality of life – consideration: age component. - Societal moral issue – over diagnosis – over treatment |
| Accountability | Auditability should be ensured throughout the AI system's life cycle to ensure responsible operation, especially regarding fundamental rights and security. It highlights the significance of comprehensive impact assessments during development and beyond. Decision-makers are accountable for balancing requirements and ethical principles. | Principle of fairness | - Societal tipping point to entrust certain decisions to AI - Doctor remains accountable (in contrast to e.g., developers) - To be legally established - Increased patient responsibility/accountability - No increased patient responsibility - Contributing as a patient – shared decision-making - AI as 2^nd^ opinion - AI as additional expert, NOT 2^nd^ opinion |
| Shared decision-making and patient-doctor relationship | Topic added by the authors for patients to reflect on in addition to the seven EGTAI requirements. | - | - Patient is responsible for their own health (lifestyle) - AI – patient autonomy is compromised - Doubts and difficulties should always be discussed with patient - AI – patient must explicitly consent with AI outcome – AI must not decide on patient’s behalf - AI informing patient alongside MD - Patient inquiring with AI (only in absence doctor) – track record and minimal explanation for recommendation rationale) |
| Trust | Topic added by the authors for patients to reflect on in addition to the seven EGTAI requirements. | - | - Hospital acts in patient’s best interest - Clinical trial AI outcome, chance of success must be substantial - Human oversight (over treatment) initially required, trust must develop - More understanding of human error than computer error - Humans make mistakes but computers even more - AI must use be disclosed to patient - Does AI consider personal context? - Differentiate between collective and individual ‘good’ – ‘what does big data say about me?’ - Suitability AI depends on the severity of the condition - AI comparisons (similar) to other tech (smartphones/car computers) - Patient trust in AI makes patient accountable (inappropriate) - AI advice in addition to doctor would be reassuring - Professional to fall back on (as a patient) |
| Question mark | The option for participants to suggest a topic themselves, which they felt was inadequately addressed by any of the other options. |  | - Emotional component (empathy) - Doctor to ensure patient autonomy and integrity - Legal accountability - Religious considerations - Financial considerations (society/collective - rising demand) - Costs should not stand in the way of health - Insurers potentially boosting AI through coverage |
