# Supplementary material for: Ethical use of artificial intelligence to prevent sudden cardiac death: an interview study of patient perspectives
Source: BMC Med Ethics. 2024 Apr 4;25:42. doi: 10.1186/s12910-024-01042-y (PMC10996273; doi:10.1186/s12910-024-01042-y)
Supplement: Supplementary file 4 — Supplementary Material 4 [file 12910_2024_1042_MOESM4_ESM.docx]

| **Requirements** | **Level of importance** | | **Scen. 1** | **Scen. 2** | **Scen. 3** | **Scen 4.** |
| --- | --- | --- | --- | --- | --- | --- |
| **Hum. Ag.** | Very important | | 18 | 16 | 17 | 16 |
|  | Important |  | 5 | 6 | 6 | 6 |
|  | Less important | | 2 | 2 | 1 | 2 |
| **Tech. Rob.** | Very important | | 12 | 12 | 15 | 4 |
|  | Important |  | 7 | 10 | 7 | 7 |
|  | Less important | | 5 | 2 | 2 | 13 |
| **Privacy** | Very important | | 5 | 4 | 7 | 8 |
|  | Important |  | 4 | 11 | 10 | 7 |
|  | Less important | | 15 | 9 | 7 | 9 |
| **Transp.** | Very important | | 13 | 14 | 15 | 16 |
|  | Important |  | 11 | 8 | 8 | 6 |
|  | Less important | | 0 | 2 | 1 | 2 |
| **Diversity** | Very important | | 7 | 8 | 8 | 9 |
|  | Important |  | 12 | 7 | 7 | 6 |
|  | Less important | | 5 | 9 | 9 | 9 |
| **Societ,** | Very important | | 1 | 2 | 3 | 4 |
|  | Important |  | 10 | 9 | 8 | 7 |
|  | Less important | | 13 | 13 | 13 | 13 |
| **Account** | Very important | | 6 | 8 | 9 | 10 |
|  | Important |  | 12 | 10 | 11 | 10 |
|  | Less important | | 6 | 6 | 4 | 4 |
| **Trust** | Very important | | 24 | 22 | 24 | 24 |
|  | Important |  | 0 | 2 | 0 | 0 |
|  | Less important | | 0 | 0 | 0 | 0 |
| **Shared d.** | Very important | | 18 | 19 | 19 | 16 |
|  | Important |  | 4 | 4 | 4 | 4 |
|  | Less important | | 2 | 1 | 1 | 4 |

**Appendix 4. EGTAI Categorization table**
